# On Diseases of the Bladder and Prostate Gland

**Published:** 1840-07

**Authors:** 


Art VI.—*On Diseases of the Bladder and Prostate Gland. With Plates.* By William Coulson. *Second Edition, greatly enlarged.*—*London*, 1840. 8vo, pp. 258.

We gave a brief notice of the first edition of this work in our Sixth Volume (p. 509), to which we beg to refer the reader. It is, however, but justice to the author to state that the present edition is not only "greatly enlarged," as stated in the title-page, but greatly improved also. It is now, indeed, a pretty complete manual of the important class of diseases of which it treats, and cannot fail to be highly useful to all junior practitioners, and even to those of older date who have not access to large libraries or have not kept their knowledge quite on a level with the progress of surgical science. Besides improving every subject, and illustrating the whole with plates and woodcuts, the author has added five new chapters; viz. one on the urine, one on spasm of the bladder, and three on the diseases of the prostate. Among other alterations we observe that Mr. Coulson has changed the title of the fourth chapter from "sub-acute" to "chronic" inflammation of the mucous membrane of the bladder, and we think judiciously,—*chronic*, although necessarily still indefinite, being certainly a better understood term than the other, while it is free from the fault of seeming to convey more information than it really does. In reading this chapter again we see that in our former article we were led into a mistake by this indefiniteness of the term sub-acute, so as to question whether the author had noticed the chronic form of inflammation at all in his former edition. This chapter, as enlarged in the present edition, gives a very excellent account of this very troublesome affection.

We once complained of Mr. Coulson for bringing his books out too fast: we shall not do so any more if he proves to us, as he has done in the present, that he industriously devotes his time to his own improvement and the promotion of sound surgical knowledge.

